# Qualitative evaluation of a preventive intervention for the offspring of parents with a history of depression

**DOI:** 10.1186/s12888-019-2273-6

**Published:** 2019-09-18

**Authors:** Nathalie Claus, Lisa Marzano, Johanna Loechner, Kornelija Starman, Alessandra Voggt, Fabian Loy, Inga Wermuth, Stephanie Haemmerle, Lina Engelmann, Mirjam Bley, Gerd Schulte-Koerne, Belinda Platt

**Affiliations:** 1 Department of Psychology, Clinical Psychology and Psychotherapy, Ludwig-Maximilians-Universität München, Leopoldstr. 13, 80802 Munich, Germany; 2 Department of Psychology, Middlesex University London, The Burroughs, Hendon, London, NW4 4BT UK; 3 Department of Child and Adolescent Psychiatry, Psychosomatics and Psychotherapy, University Hospital Munich, Nussbaumstr. 5a, 80336 Munich, Germany

**Keywords:** Prevention of depression, Offspring of depressed parents, Qualitative evaluation, Mixed-methods approach

## Abstract

**Background:**

Meta-analyses of randomised controlled trials suggest that psychological interventions to reduce children’s risk of depression are effective. Nevertheless, these effects are modest and diminish over time. The Medical Research Council recommends a mixed-methods approach to the evaluation of complex interventions. By gaining a more thorough understanding of participants’ perspectives, qualitative evaluations of preventive interventions could improve their efficacy, longevity and transfer into clinical practice.

**Methods:**

18 parents and 22 children who had received a 12-session family- and group-based cognitive-behavioural intervention to prevent youth depression as part of a randomised controlled trial took part in semi-structured interviews or a focus group about aspects which had been perceived as helpful, elements they were still using after the intervention had ended, and suggestions they had for improving the intervention.

**Results:**

The chance to openly share and discuss their experiences of depression within and between families was considered helpful by both children and parents. Children benefitted the most from learning coping strategies for dealing with stress and many still used them in everyday life. Parents profited mostly from increasing positive family time, but noted that maintaining new routines after the end of the intervention proved difficult. Participants were generally content with the intervention but commented on how tiring and time consuming it was.

**Conclusions:**

Managing parents’ expectations of family-based interventions in terms of their own mental health needs (versus those of their children) and leaving more room for open discussions may result in interventions which are more appealing to participating families. Increasing intervals between sessions may be one means of improving the longevity of interventions.

**Trial registration:**

The original RCT this evaluation is a part of was registered under NCT02115880.

## Background

### The role of parental depression as a risk-factor for youth depression

One of the most detrimental risk factors for depression in children and adolescents is parental depression [1, 2]. Compared to children of parents who have never experienced mental illness, offspring of parents who have been depressed have a two- to threefold greater risk for major depressive disorder [3–10]. This equates to a 50% risk for developing depression by the age of 20 in children with one parent who has been depressed, which increases to 70% if both parents have experienced depression [11–14]. Episodes of depression during childhood and adolescence are not only associated with short-term negative outcomes such as negative educational achievement and adverse social relationships [2, 15], but are also strongly associated with depression, substance misuse and suicidal behaviour later in life [16]. Although effective treatments for adolescent depression do exist [17], the personal, social and economic burden of depression [18] means that developing effective preventive interventions for the offspring of parents with depression is a major public health priority [1]. Meta-analyses demonstrate that youth depression is indeed preventable [19, 20], although the evidence base for interventions for offspring of parents with depression specifically is less well established (see Loechner et al. [21] for a review of preventive interventions for children of parents with depression).

### Preventive interventions for the offspring of parents with depression

A number of biological (e.g. genetic), psychological (e.g. learnt maladaptive coping strategies for dealing with stress) and environmental (e.g. exposure to parental conflict) factors are proposed to underlie the elevated risk of depression in offspring of parents with depression [22]. These potential mechanisms have formed the basis for a number of preventive interventions [23–27] which contain one or more of three ‘key ingredients’: i) *psychoeducation* about the symptoms, cause, and course of their parents’ depression aims to increase children’s sense of security and control and reduce their sense of anxiety about and responsibility for their parents’ wellbeing, ii) teaching adaptive *coping strategies* (acceptance, distraction, positive thinking, positive activities) for dealing with stressful situations, and iii) *parental training* designed to reduce the negative impact of parental depression on the child. These preventive interventions have been evaluated in randomised controlled trials (RCTs) and together, they show small to moderate effects on the onset of depression (RR = 0.56; NNT = 4.28), at least in the short-term [21]. However, the relative benefits of the three key elements are unclear, and there remains a need to further develop these interventions to improve their efficacy and longevity. In addition, little is known about how transferable these interventions are from the research setting to clinical practice.

### The “GuG auf” intervention

The “Raising Healthy Children” intervention developed by Compas and colleagues [24, 28] was translated and adapted to German culture (“GuG auf - Gesund und Glücklich aufwachsen!”) and is currently being evaluated in an ongoing randomized controlled trial [29]. The group- and family-based programme offers eight weekly and four monthly booster sessions for four to five families, including homework assignments. It combines psychoeducation for the whole family (first three sessions plus booster sessions) with stress coping strategies for children and positive parenting skills for parents in five additional separate sessions. Sessions last two hours and are conducted by qualified study members (trainee psychiatrists, psychologists, doctoral students) who are trained and supervised by a post-doctoral researcher. For this study, sessions took place in the afternoon, at the Department of Child and Adolescent Psychiatry at the University Hospital Munich, Germany.

### The benefits of qualitative research

Existing evaluations of prevention programmes for the offspring of parents with depression have largely been conducted in the form of controlled trials. The RCT is often considered to be the “gold standard” in the assessment of psychological interventions [30–32] and enables observed effects to be attributed to the intervention specifically [33, 34]. However, as Midgley and colleagues [32] identify, RCTs often lack external validity in the context of psychotherapy evaluation [35], resulting in difficulties in translating RCT findings into effective changes in clinical practice [36]. One key element of *quantitative* research is its standardised and predetermined measures, which promote reliability and validity, but can miss out on issues important to participants that go beyond symptom-relief and were not necessarily expected to be relevant [37]. Midgley et al. [32] describe these issues as easier to penetrate via *qualitative* methods (e.g. open-ended interviews), which are explorative rather than hypothesis-driven [37].

A mixed-methods approach that combines quantitative and qualitative measures [38] as recommended by the Medical Research Council Guidance for complex interventions [39] is becoming increasingly common in the evaluation of psychotherapy [40–42] and depression treatments specifically [32, 43–46]. Incorporating participants’ individual experiences has already been performed in the evaluation of universal prevention programmes [47–50]. Since qualitative methods have been deemed particularly suitable for understanding the process behind therapeutic change rather than outcomes [51, 52], they may offer insight into the relative effectiveness of the three key elements of preventions for offspring of parents with depression mentioned above. As far as we are aware, only one qualitative study of preventive interventions for this target group has been conducted. Pihkala & Johansson [53] used qualitative interviews to explore parents’ (but not children’s) motivations for taking part in the psychoeducative Family Talk Intervention [12], as well as factors which were perceived as facilitating prevention success.

### The current study

This qualitative study complements quantitative data being collected in an ongoing RCT study of a preventive intervention for the offspring of depressed parents [29] and is as such to be read in the broader mixed-methods context of the evaluation. Forty out of 80 children and parents who had participated in the preventive intervention took part in semi-structured interviews or a focus group about their experiences of the intervention. The key aims of the study were to understand which elements of the intervention participants found particularly helpful and how they thought the intervention could be improved, as well as how well participants managed to transfer what they had learnt into everyday life. This approach was adopted to find a balance between gaining feedback on areas of key interest to the team as well as allowing participants the opportunity to raise additional points and concerns in their own words rather than a survey with a limited range of response options. The study was designed to inform the future development of this intervention specifically as well as future preventive interventions for offspring of parents with depression and their implementation into clinical practice more generally.

## Methods

### Design

Participants who partook in the intervention (described above) were asked to participate in qualitative interviews or a focus group upon completion of the 15-month intervention period. One-to-one interviews followed a semi-structured schedule (Appendices 1 and 2), which was an adaptation of the topic guide developed for the “TARGET” group at King’s College London (Patrick Clark, personal communication) to explore topics relevant to the process of improving the prevention programme. Additionally, a focus group was conducted in order to discuss the same topics in a group setting with both parents and children. As there were similarities in the content categories emerging from the focus group and interview data, these are presented together. This study is reported in accordance with the COREQ guidelines [54], the checklist is included in Additional file 1.

### Participants

In qualitative research power calculations are not appropriate and the required sample size relies on the concept of “data saturation” [54, 55], meaning the point at which no new information is being generated by adding new data. In advance, this was assumed to be the case after around 20 to 30 interviews. However, data saturation wasn’t measured separately.

Families who participated in the preventive intervention consisted of at least one parent with a diagnosed episode of depression according to DSM-IV-TR criteria [56] during the child’s lifetime (either current or in remission) and at least one child aged 8–17 years who had no lifetime history of psychiatric illness. Psychiatric status was assessed using the Diagnostic Interview of Psychiatric Disorders (DIPS [57]) and the Diagnostic Interview of Psychiatric Disorders for children and adolescents (K-DIPS [58]). The participation of the second parent was optional. Of the 25 families who had completed the intervention at the time of data collection, 16 families were invited to participate in an interview or focus group about their experiences of the intervention. They were selected because they had been the first to enroll in the intervention and had completed it four to 13 months prior to this study; the only exception were families taking part in the focus group who took part immediately after their last group session since this was the most practical way of coordinating families. The remaining nine families were not contacted because they had only completed the intervention within the past few weeks and the study was partly designed to assess how able families were to implement the intervention contents into their lives in the mid-term. Fifteen families (18 adults and 22 children) agreed to answer questions about their experiences. This sample (described in more detail in Table 1) includes 17 parents with a history of depression, 22 children (one or two children per family), and one partner that did not suffer from depression. Age ranged from 37 to 54 years for parents and from nine to 17 years for children, with gender distributed relatively equally for both parents and children. All participants were of German nationality (good command of the German language was an inclusion criterion for the intervention) and the vast majority of parents (93%) were living together. T-tests and Chi^2^-tests revealed that this sample did not differ significantly from the full sample of participants who had received the intervention at the time (*N* = 80) in any of the variables reported below (all Ps > 0.05).

**Table 1:** Demographic information about the interview and focus group sample

Primary parent^a^	n	Mean (SD)	Min-Max	%
Age	15	47.73 (5.27)	37–54	
Gender (f/m)	15			40/60
Highest level of Education (Secondary school/A-levels/undergraduate degree/PhD)	15			6/27/47/20
Parents living together (y/n)	13/1			93/7
Nationality: German (y/n)	13/0			100/0
Depressive episode at start of intervention (yes/no)	11/4			73/27
Number of previous depressive episodes	12	7.10 (5.10)	1–15	
Depression severity (BDI prior to intervention)	15	20.47 (10.16)	1–40	
Treated for depression prior to intervention (y/n)	13/1			93/7
Partner^b^				
Age	2	42.50 (2.12)	41–44	
Gender (w/m)	3			67/33
Highest level of Education (Secondary school/A-levels/undergraduate degree)	3			33/33/33
Children				
Age	22	13.09 (2.41)	9–17	
Gender (w/m)	22			50/50

aThe parent with a history of depression through which the family fulfilled the study eligibility criteria

^b^Two of the three partners had experienced an episode of depression themselves

### Procedure

Translated schedules for the parent and child interviews and the focus group can be found in Appendices 1, 2 and 3 respectively. They included the same key topics: Had parents and children learned anything new and if so, how much of that knowledge were they still using in everyday life; how had their reactions to stress changed; how had the topic of depression been addressed in the family before the intervention as opposed to during; which logistic factors facilitated, which hindered prevention success. However, the interview structure remained flexible in order to allow participants to raise additional points and concerns. All participants were informed that these feedback interviews were voluntary and that they could withdraw their answers at any time, but were asked to be as honest as possible to help improve the intervention for future participants.

#### Interviews

Thirty-five participants were invited to take part in interviews either in person as part of their final assessment session for the RCT (15 months after the study began) or via telephone afterwards. Whenever possible, interviews were conducted face-to-face in the Department of Child and Adolescent Psychiatry (25 out of 35 interviews) and where this was not possible via telephone at a time of day suggested by the participant (10 out of 35 interviews). Interviews were conducted by four undergraduate Psychology students, the majority by NC as part of a bachelor’s thesis project. Both parents and children were interviewed individually without anyone else present in the room, with participants sitting at a table across from the interviewer and the audio recording device placed on the table. Participants were asked the same questions from the semi-structured interview schedule in order to ensure the same topics of interest were covered, with the option of sharing any additional experiences that had not directly been addressed at the end of the interview. They were given as much time as needed and encouraged to honestly share everything they considered relevant to the improvement of the intervention. Interviews lasted between ten and 40 min, with the parent interviews typically being longer and more elaborate than those with the children.

#### Focus group

One focus group was conducted by NC with five additional participants who had not been interviewed (three parents and two children) to discuss the same questions in a group setting. The focus group was included on the assumption that a group discussion rather than a one-on-one interview would allow for disagreement between participants and thus potentially more diverse responses and richer data. The focus group lasted approximately 30 min.

### Data analysis

The interviews and focus group were recorded with a voice recorder (with the interviewer additionally taking notes during the interviews), anonymised, and subsequently transcribed verbatim into MS Word. Some of the audio files of the interviews (*N* = 13/35) were not saved correctly and were analysed based on the interviewer’s written notes (bullet points) alone. Quotes in the Results section are identified as participants’ own words or paraphrased interview notes.

A deductive qualitative content analysis was carried out by one coder, following the three phases of analysis recommended by Elo and Kyngäs [59]. A categorisation matrix which covered all presupposed matters of interest was developed and the data was coded for correspondence with these categories [60] by reading and re-reading all transcripts and interview notes. An unconstrained matrix was used in order to be able to create sub-categories based on participants’ replies within the bounds of the presupposed categories [61, 62]. A coding frame was developed to facilitate coding of interview transcripts using the software NVivo [63]. Only manifest content was analysed, with single interviews as unit of analysis [64]. Final identification of categories was based on consensus discussion between members of the research team (NC, BP, LM). Key categories were then described and illustrated with quotes, and then related to each other and back to the research question. Answers are reported below grouped around those categories, with all quotes translated into English from their German original.

### Reflexivity

The process of data analysis is influenced by the researcher’s personal perspective, so a degree of “reflexivity” is required [65]. The majority of interviews were conducted by NC (B.A. in Communications and undergraduate Psychology student at the time) who knew the intervention well and had occasionally delivered sessions. This involvement may have put them in a better position to understand and discuss the content of participants’ feedback but may also have led them to perceive participants’ responses less critically than someone independent to the project. The fact that some families knew NC may have led them to be less honest or equally to have opened up more easily. Having conducted qualitative research as part of a dissertation before, NC did their best to encourage honest feedback and minimise the impact of social desirability.

## Results

Across the various broad topics in the interview script, numerous strengths and limitations of the GuG auf! intervention could be identified (Table 2). Where responses differed between parents and children this is described; responses from parents with a history of depression and their partners are reported together. Additionally, summaries of key topics including quotes are provided.

**Table 2:** Participants’ perspectives on strengths and limitations of the intervention, with *N* = 18 parents and *N* = 22 children (*N* = 40)

Topic	Strengths	Limitations	Neutral comments
General acceptability	Generally “nice”, “important”, “helpful” or “encouraging” (*N* = 21)	Very tiring / time consuming (*N* = 17); most contents already familiar or self-evident (*N* = 8); expectations not met (*N* = 2)	Parents: Make future participants aware of time consuming nature (*N* = 6), of focus on children (*N* = 5), that they may benefit more if not currently depressed (*N* = 2)
	Parents: Would recommend intervention to other families (*N* = 18); interactivity (*N* = 6)		
Motivation for participating			Contributing to a research project (*N* = 3)
			Parents: Supporting their children (*N* = 10)
Talking about depression	Opportunity to exchange experiences with other affected families (*N* = 10)		Children: Talked about depression with friends through intervention (*N* = *11*)
	Children: Positive experience (*N* = 21); most helpful aspect of intervention (*N* = 8)		
	Parents: Easier to broach the subject of depression (*N* = 9); reduced guilt (*N* = 5); external source of information (*N* = 3)		
Children’s knowledge of depression (children’s view)	Gained new knowledge from intervention (*N* = 10)	Not learned anything new (*N* = 2)	
Children’s coping with stress (children’s view)	Most helpful aspect of the intervention (*N* = 15); more in control of thoughts (*N* = 13); more relaxed now (*N* = 8); gained new knowledge (*N* = 6)	Not learned anything new (*N* = 7); could not control feelings any better (*N* = 7)	
Parenting skills	Family activities increased as a result (*N* = 8); practised parenting strategies (*N* = 6); gave more attention and praise (*N* = 3); questioned automatic parenting behaviour (*N* = 2); structure and repetition of already familiar ideas (*N* = 2)	Children: Did not believe parenting style had changed (*N* = 11)	Had no prior expectations of learning anything new (*N* = 2)
Implementation in everyday life	Intervention implemented (*N* = 10)	Not automatic enough (*N* = 8)	
	Children: Implemented stress coping strategies (*N* = 14) and would recommend them to a friend (*N* = 13); initial response to stress improved (*N* = 9)		
	Parents: Family activities implemented (*N* = 4); increased self-reflection (*N* = 4); parenting strategies (*N* = 2)		
Logistics	Size of groups (*N* = 32); time slot (*N* = 32); location (*N* = 34)	Parents: Too intense (*N* = 6); too much homework (*N* = 2); not enough information and challenges (*N* = 3); did not adequately involve the whole family (*N* = 3)	Online content welcome but not necessary (*N* = 5); consistency of group leader important (*N* = 3)
	Children: Group separation (*N* = 15)		
	Parents: Amount of information provided (*N* = 12)		

### General acceptability

Although the most commonly named disadvantage to participating in the intervention was how tiring and time consuming it was (*N* = 17) due to weekly sessions and homework assignments, participants generally displayed a positive attitude towards the prevention intervention and the effort was deemed to be ultimately worthwhile:

"It was encouraging, I think. It gave me courage, that you shouldn't immediately give up." (Child of family 14, transcribed)

For most of them, the intervention represented a valuable first step in starting a conversation:

"I wouldn’t say we’re all irrevocably super happy with each other, but it definitely paved a way." (Parent of family 5, transcribed)

All parents (*N* = 18) would recommend the intervention to other families, albeit with some caveats (see Table 2):

"If you've got someone like that [currently in a depressive episode], then better tell them wait, wait a little bit, because they can't take in much (...). I'll be completely honest, if it'd been three or four months later, when my psychiatrist had stabilised me a bit, maybe it would've been better." (Parent of family 9, transcribed).

Six parents praised the interactive character of the intervention as particularly helpful, including role-plays, which increased their active interest in the content. Some participants (*N* = 8), however, described most of the intervention’s contents as already familiar or self-evident:

"Some advice was simply already, uh, being practised in the families. And if participation's voluntary, then it'll be mostly people who are already dedicated to their children, who don't just not care about their children." (Parent of family 10, transcribed).

#### First memories

When asked which elements of the intervention they could best recall, both children and parents named the stress coping strategies (*N* = 13), referred to in the intervention as “A’APP”:

"Even though I admittedly don't always immediately know what it means, there's of course these abbreviations, this A'APP. Things like that just stick." (Parent of family 14, transcribed).

### Talking about depression

#### Before the intervention

Relatively few children were completely unaware of their parent’s depression before taking part in the intervention (*N* = 6). Over half of them (*N* = 14) said they were definitely aware before but often did not know much specifically and could not recall explicitly talking about it with their parents.

Five parents expressed reluctance to bring up the topic with their children on their own or in detail because of the potential impact it would have on their children:

"Before, you actually, I mean for my part tended to keep it secret, because you didn't want to put a strain on the child." (Parent of family 5, transcribed).

#### After the intervention

One aspect of the intervention that was perceived to be very helpful by both parents and children was the chance to exchange experiences with other affected families (*N* = 10):

"Because you hear a lot about, well, this percentage of the population feels the same, but you uh, it's something different when those people are sitting in front of me and sharing their everyday experiences." (Parent of family 5, transcribed).

In contrast to parents’ apprehensions (see above), most children (*N* = 21) perceived talking about their parents’ depression as a positive experience. The parents’ experiences were similarly positive: Nine of them said the intervention had made it easier to broach the subject within the family:

"Simply the, uh, distance to the topic was kind of gone afterwards and that helps of course, yes, I have to admit that helped me an incredible amount." (Parent of family 8, transcribed)

Three said the information coming from outside of the family had helped to establish common knowledge and hear how their children felt about it. A perhaps surprising number of parents (*N* = 5) quickly brought up the question of guilt as well, explaining that it was a particular relief to have someone else tell their children that their parents’ depression was not their fault:

"Even if they know that already, it’s good if someone else tells them as well, and even more so if they don’t know." (Parent of family 6, transcribed)

Two of them argued with their own family history:

"I remember when I was fourteen, fifteen, I wasn’t aware of how my mother was doing. And if someone had said – which I actually found trivial now – she isn’t this grumpy because of you (…), that probably would have helped me a lot." (Parent of family 5, transcribed)

#### Children’s knowledge of depression

Ten children said they now knew more than before the intervention about what depression is:

"That depression is curable and that people still, you know, are normal. That they're just not mentally disturbed, numb people now." (Child of family 12, transcribed).

### Evaluating new skills

#### Children’s coping with stress

Eight children said the intervention had helped them cope with stress and helped them to feel more relaxed. Thirteen children said that they could better control or influence their own *thoughts* one way or the other after the intervention. Two said they generally felt more in control since they had practised acceptance and staying calm:

"I used to think: $h!t! Now I think: Stay calm for now." (Child 1 of family 1, written notes).

Children were a little more sceptical about being able to influence their own *feelings*, with seven of them saying they could not influence their feelings any better now. Of note, a lot of children, especially the younger ones, seemed to have difficulty differentiating between their thoughts and their feelings.

#### Parenting skills

Eight parents specifically called being encouraged to make time for more family activities helpful:

"To really take the time for the whole family. And not just like, 'yes, we could sometime', but actually planning it in more detail." (Parent of family 10, transcribed).

Six parents specifically indicated clear parenting rules and practising how to implement them as helpful:

"Being able to try out such a reward system for a bit, or simply implementing certain parenting strategies that I’d heard of before in theory but just never really officially tried out." (Parent of family 5, transcribed)

However, half of the children (*N* = 11) did not believe their parents had changed anything about their parenting style.

#### Implementation in everyday life

Ten participants (3 parents and 7 children) said they were able to implement the intervention into everyday life. The majority of the children said they still used some or all of the stress coping strategies (*N* = 14), predominantly distraction (*N* = 7). Nine children specifically mentioned the stress coping strategies as their go-to when faced with a stressful situation, and thirteen would recommend them to a friend looking for advice:

"I think that doesn't just help people who, I don't know, have depression, but all people who get stressed sometimes." (Child 1 of family 5, transcribed).

Eight participants said they still used the learned strategies sometimes, but the process was not automatic enough by the time the intervention was over:

"It's all such a shame (...). For some time we kept doing it, but then it kind of slipped away. You somehow forget about it in your daily routine." (Parent of family 13, transcribed).

### Logistics

The majority of participants were happy with the size of the group (*N* = 32), the time slot for their sessions (*N* = 32), and the location (*N* = 34). Children were generally content with being able to attend both children-specific sessions and sessions shared with their parents (*N* = 15).

Some parents, however, suggested changes in future, wishing either for a less intense intervention (*N* = 6) with less homework (*N* = 2) or for more information and challenges (*N* = 3). Four parents admitted they had been very negligent with the homework, one of which stated they could have profited more if they had done all of the homework:

"But I thought, better to continue without the homework than have great intentions and then say, I can’t do it, and quit." (Parent of family 6, transcribed)

Another three would have liked for the intervention to strive to better involve the whole of the family, including the partner who did not actively partake.

Without direct prompting, some parents emphasised the group leaders’ central role to the intervention’s perceived effectiveness, three of which expressed regret that their group leaders had alternated so much and so their group had somewhat lacked consistency.

The focus group addressed the potential of future online content and how participants would feel about it. All participants were happy with printed materials, but would not have minded additional online content.

## Discussion

Interventions designed to prevent depression in the children of parents with a history of depression have been largely evaluated in (randomised) controlled trials. Since the effect sizes of these interventions are modest to moderate at best [21], there is a clear need to investigate which aspects of these complex interventions work best and how existing interventions might be modified to address limitations. Qualitative methods may be better placed to explore these issues and to investigate factors which may enhance or impair implementation of these interventions into practice. To complement quantitative data collected from an RCT study of a preventive intervention for children of parents with depression [29], the present study reports on participants’ subjective experiences of their participation in the intervention. Our findings specifically include feedback from both parents and children as most studies to date have neglected the children’s perspective on intervention success, despite evidence that relying on parental reports alone, particularly depressed parents’ reports of their children’s emotional or behavioural problems, is likely to result in distorted data [66].

### Interpretation of findings

Acceptability of the intervention appears to be relatively high: All 18 interviewed parents would encourage families in a similar situation to partake in an intervention like this. The fact that the majority of participants had experienced the intervention as helpful appears to be in line with the original RCT investigating the benefits of this particular family group prevention programme [28] as well as meta-analytic results which show significant, albeit small preventive effects in offspring of depressed parents [21]. Further findings of the study are discussed in relation to the three key components of the intervention mentioned in the introduction: psychoeducation, coping strategies and parenting strategies.

The central aspect that was considered helpful by the majority of both children and parents was sharing their experiences of depression within the family (i.e. *psychoeducation*). After the intervention children felt like they better understood how depression affected their own parents personally and parents felt relieved that the topic was now “out in the open” and no longer a source of discomfort or guilt. This is in line with Beardslee and colleagues [11, 67–69] who argue that interventions which enable an open conversation about depression within the family can be enough to help prevent child depression. According to the study by Pihkala and colleagues [53], the inability to explain their depression by themselves and the need for professional support and mediation is common among parents with a history of depression. Importantly, children described discussing their parent’s depression within the family as a distinctly positive experience and did not view it as distressing, but beneficial. This contradicts the frequently reported parental fear of unnecessarily burdening children with details about their mental illness and wanting to protect them from further stress [70, 71], which not only tends to hinder open discourse within the family but also constitutes another hurdle for participation in prevention programmes. Based on these findings, this fear appears to be unfounded.

The learning content that stuck with participants the most were stress *coping strategies*. The children specifically appeared to benefit from having a toolbox for dealing with stressful situations, many of them citing this as the most helpful aspect of the intervention. Using these strategies in their everyday life, they perceived themselves to be more in charge of their own thoughts and feelings, effectively improving their self-regulation skills. This positive feedback from the children’s perspective adds to previous works in that it suggests that elements of cognitive-behavioural therapy (CBT) may be a valuable contribution to more basic psycho-educative interventions because they make children feel more in control. There was no single coping strategy that was reported as being more or less helpful, although this question was not posed directly to children.

A lot of content regarding *parenting strategies* (e.g. positive family time) seemed self-evident to some parents, perhaps due to high motivation levels of the selective sample and/or their experience of psychotherapy. Nevertheless, many of them still felt like they had benefited from the intervention by consciously implementing, practicing and repeating certain contents. This implies that behavioural activation played a particularly important role in the intervention.

While most participants were content with the amount of information presented in the intervention and found its structure and regularity helpful, a few would have preferred more and others less. Most agreed, however, that sessions often felt rushed. Indeed, the group leaders often had trouble covering the contents of the manual because parents particularly had a need to talk more intensively about their personal experiences at home. This may reflect the fact that not all of them (although encouraged) were currently receiving psychotherapy and/or the fact that the intervention, delivered as part of an RCT, was heavily manualised. Participants whose group leaders had alternated a lot expressed regret about their group lacking consistency. Focus group participants seemed indifferent towards the option of potential online content in future versions of the intervention.

## Conclusions

There are several impulses that could be taken from the current study in order to enhance the implementation of such interventions into practice, to improve their acceptability and longevity, and to guide future research in this field.

### Implementing interventions into practice

Parents’ expectations of preventive interventions should be managed appropriately in advance. Although the researchers gave participants a detailed description of what to expect from the intervention prior to study inclusion, the majority of participants described being surprised by the time-consuming nature of the intervention and the amount of active participation required, especially regarding homework. Furthermore, not all parents were aware that the intervention was aimed mainly at supporting the children rather than dealing with the parents’ depression. A better, more precise understanding of what the intervention provides as well as demands could reduce feelings of disappointment or being overwhelmed. Since most parents who explained their motivation for taking part said they did it to protect their children, this could be used to promote the intervention. Intervention promotion could also include quotes from participants who explained how the group setting and being able to share their experiences helped them overcome their initial anxiety and made it easier to address the subject in the family. Crucially, recruitment should make use of the finding that children were not distressed by the confrontation with their parent’s depression, as is often feared by parents, but instead viewed talking about it as a positive experience.

Just as importantly, parents’ current well-being should be taken into consideration when assessing suitability for preventive interventions. Similarly to findings reported by Pihkala and colleagues [53], those parents who had participated shortly after suffering a depressive episode did not feel like they could profit from such an interactive intervention, a finding supported by an RCT study of the same intervention [24]. Simultaneous psychotherapy treatment might also be further encouraged, given the finding that parents in the current study hoped for more time to discuss issues related to their own depression.

### Improving acceptability and longevity

Based on the finding that parents expressed differing expectations of the intervention, wished more flexibility from the intervention and considered a lot of contents to be self-evident, interventions like this might generally need to be more flexible regarding the contents they provide. For example, the promotion of regular positive family time was considered to be either particularly helpful or redundant by participants, depending on how established it had already been within families before joining the intervention. Participants’ frustration could be minimized by using the first few sessions to gauge the needs of individual groups and adapt contents to them.

Modifying the frequency of sessions could also help improve long-term gains from the intervention. Several participants expressed regret that a lot of knowledge remained only passive or was “not automatic enough” by the time the intervention ended. Better preparing them for the end of the intervention may be easier if the time between the last few sessions increases step by step, to make the break between “during” and “after” the intervention feel less sudden.

Finally, a lot of participants only mentioned the term “homework” in a negative context and expressed regret or frustration over the amount, but at the same time would have liked more training opportunities and for their progress to persist. Thus, homework might be framed more consistently as “training” and considered a positive learning opportunity. Additionally, the potentially negative impact of fluctuating group leaders on how acceptable the intervention is should not be underestimated.

### Future research

The present work has shown that qualitative data can offer valuable additional information about what kind of an impact preventive interventions like this can have on participants’ everyday lives and which elements are perceived as particularly conducive or obstructive to prevention success. Researchers investigating prevention effects should adopt a mixed-methods approach as standard procedure in the future to avoid the “implementation gap” [72] so often associated with strictly quantitative evaluations. It might be worth considering using interviewers that are not part of the project and so have fewer preconceptions about its merit. Lastly, it would be interesting to further investigate any difference in intervention effectiveness and acceptability for families who participated with one parent only compared to both parents taking part in the intervention.

### Strengths & Limitations

A strength of this work is its mixed-methods approach, in which quantitative data collected in an RCT are supplemented with qualitative feedback. To our knowledge, it is the first qualitative evaluation of a selective prevention intervention for families with depression that takes into account both the parents’ and the children’s view. The explorative nature of this work allowed us to investigate participants’ perceptions of the strengths and weaknesses of the intervention which might not have been addressed in the quantitative evaluation. Furthermore, the findings have additional implications for future research as well as implementing such interventions into clinical practice. Since effects on mediators and moderators are inconsistent [73, 74] and mechanisms of prevention interventions remain unknown, taking the perspective of the recipients of those interventions is essential in order to increase modest preventive intervention effects [19–21]. We were able to collect data from 40 family members in total and also to conduct a focus group in order to garner richer data. However, it is important to keep in mind that due to the qualitative study design, the data are not intended to be representative.

A limitation is the nature of the sample: Despite a family history of depression, all study participants had taken part in a time-consuming intervention. This indicates a particularly motivated sample. In addition, families were mostly German and had a high socio-economic background. Consequently, the intervention might be experienced differently by families from different social and cultural backgrounds, who might have different parenting values and/or financial and time constraints. These families are often those who are neglected regarding psychosocial support and have limited access to mental healthcare [75]. Furthermore, the inclusion of children and adolescents resulted in shorter interviews and focus group and thus less data; it also meant participants could often only be reached by telephone and so many of the interviews were conducted via telephone rather than in person. Another limitation was the structure of the interview schedule, which was not sufficiently oriented toward the younger participants and made it difficult to yield more elaborate replies. Unfortunately, some early interviews were not recorded and the interviewer instead made notes during the interview, resulting in data of a poorer quality.

Finally, we experienced difficulties organising and conducting the focus group. Of the four group families, only two could be present for the focus group due to personal time restrictions such that group discussion was limited.

### Supplementary information

**Additional file 1.** COREQ Checklist. Checklist for qualitative research papers in order to uphold high research standards and provide transparency for the readers.

## Data Availability

The datasets generated and/or analysed during the current study are not publicly available since the publication of raw data was not included in the consent forms, but all data and materials are available from the corresponding author on reasonable request.

## Appendix 1

### Interview schedule for parents (Translated from German original)

Thank you for participating! The interview is going to take 30–45 min. We ask everyone who has participated in the programme for their feedback in order to improve our programme. You can ask at any time if something is unclear.

Similar to the other sessions we are going to make an audio recording of this evaluation so we can transcribe the discussion later on. We are going to remove your names from your feedback and the group leaders are not going to find out who said what, that means that you can be absolutely honest. Things we discuss are, like in other GuG auf sessions, confidential.

Topic guide

- What do you think about the programme in general?
- What was helpful or not helpful at all?
- What do you remember best?
- Did you learn something new?
- Did you learn something that you currently use or that you would use during stressful situations in the future?
- How could these strategies help to improve the situation? Was there something you would have liked to talk about that we did not cover?
- Would you recommend the programme to a friend?
- What advantages and disadvantages did you experience as a participant of the programme?
- Was the size of the group comfortable?
- Did your daughter/son know that depression is an issue in your family before you participated in the programme?
  - ◦ If not, how was it for you to tell your child about it? Or to participate in the programme?
  - ◦ What was it like to explain your depression to your children?
- Did you feel like the programme focused on the important things for your family?
- In your opinion, how much did you get involved in the programme?
- Are you happy with the amount of information you received during the programme?
- Do you think that other families should participate in such programmes?
- Did you like the time and the location for the group sessions?

## Appendix 2

### Interview schedule for children (Translated from German original)

Thank you for participating! The interview is going to take 30–45 min. We ask every child who participated in our programme to give us feedback. This interview is going to help us to evaluate and to improve our programme. You can ask at any time if something is unclear.

Similar to the other sessions we are going to make an audio recording of this evaluation so we can transcribe the discussion later on. We are going to remove your names from your feedback and the group leaders are not going to find out who said what, that means that you can be absolutely honest. Things we discuss are, like in other GuG auf sessions, confidential.

- Did you know that your mother/father has suffered from depression before you participated in the programme?
- ○ If not, how was it for you to hear about it for the first time? (Did it make you feel sad, worried, interested…)?
- Did your knowledge about depression change?
- What do you think about the programme? (The conversations, your contributions, workbook, training, etc)
- What helped you the most/least?
- What do you remember most?
- Did you learn something new?
- Did you learn something that you now use? Or can you imagine that you would use one of the strategies during stressful situations or when you’re feeling sad in the future? How could you improve those?
- What advice would you give to your friends when they are sad or stressed?
- Do you cope with stress now in another way than before the programme? If yes, how?
- Did you tell your friends about the sessions? If yes, what do they think about it?
- Which advantages and disadvantages did you experience as a participant of the programme?
- Was the size of the group comfortable?
- Did you like the time and the location for group sessions?
- Do you think your parents have changed their parenting behaviour?
- Would you prefer a group that is only for children or a mixed group with parents?
- Do you feel like you have better control of your thoughts now? Do you think you have better control of your feelings?

## Appendix 3

### Focus group schedule (Translated from German original)

Thank you for agreeing to participate in today’s evaluation. We appreciate you taking time to share your experiences with us. This evaluation helps us to find out which parts of GuG auf^1^ worked well and what we should change in the future to support participating families best. Your comments are important because you have passed through the whole programme and you know best what it means to participate in GuG auf.

Similar to the other sessions we are going to record this evaluation on video so we can transcribe the discussion later on. We are going to remove your names from your feedback and the group leaders are not going to find out who said what, that means that you can be absolutely honest. Things we discuss are, like in other GuG auf sessions, confidential.

If there are any questions you don’t want to answer, then you don’t have to – but of course it would be nice to get as much feedback as possible.

So we don’t miss any important feedback, please make sure only one person talks at a time. There is no right or wrong, we just would like to hear your honest opinion. Everyone can answer freely, say things that come to your mind, without a fixed order. Are there any questions before we begin?

**Questions / Topics:**

- General feedback – what do you think about GuG auf now at the end of the programme?
- What was especially helpful for you? What was not helpful at all or even unnecessary?
- What do you remember best? What is the first thing that comes to your mind when you think of GuG auf?
- Did you learn something new from GuG auf?
- Is there something that you learnt in GuG auf that you currently use in everyday life when you feel stressed or in a bad mood?
- Were there any issues that were not discussed but that you would have liked to talk about? Was there anything missing?
- Did you have the impression that the programme focused on the important issues in your family?
- Would you recommend the programme to a friend?
- What were the advantages of taking part in GuG auf? Were there any disadvantages?
- What was it like in your family, did you talk about depression with each other before participating in GuG auf? Was it different talking about it in the GuG auf sessions as opposed to at home? If yes, how?
- In your opinion, how intense was your commitment to GuG auf?
- Were you happy with the amount of information you received through GuG auf? Was it too much/too little?
- Did you like the group size? Did you like the location? The time of day?
- We’re thinking about integrating the internet into GuG auf a bit more in the future. For example, filling in the homework online or providing the workbooks online. Do you think this would make sense?
- Is there anything else you would like to mention that I have not asked you about?

Thank you for your honesty!

## Abbreviations

A’APP: Acceptance, Distraction, Positive Thoughts, Positive Activities (*Akzeptanz, Ablenkung, Positive Gedanken, Positive Aktivitäten*); mnemonic device to help participants remember the stress coping strategies they learned throughout the programme
CBT: Cognitive-behavioural therapy
RCT: Randomised controlled trial
